# Computational Simulation of a Surface Plasmonic Resonance Biosensor for β2-Microglobulin Based on Electrolyte-Gated Graphene

**DOI:** 10.3390/s26092815

**Published:** 2026-04-30

**Authors:** Ghassem Baridi, Arslan Liaquat, Leonardo Martini, Federico Rapuzzi, Vito Clericò, Mario Amado, Enrique Diez, El Hadj Abidi, Maria Celeste Maschio, Stefano Corni, Yahya Moubarak Meziani, Giorgia Brancolini, Francesco Rossella, Luigi Rovati

**Affiliations:** 1 Department of Engineering “Enzo Ferrari”, University of Modena and Reggio Emilia, Via P. Vivarelli, 10, 41125 Modena, Italy; luigi.rovati@unimore.it; 2 Department of Physics, Computer Science and Mathematics, University of Modena and Reggio Emilia, Via Campi 213/a, 41125 Modena, Italy; 3 Department of Applied Physics, University of Salamanca, 37008 Salamanca, Spain; 4 Nanoscience Institute-Esse 3, National Research Council, Via Campi 213/a, 41125 Modena, Italy

**Keywords:** biosensor, surface plasmonic resonance, electric double layer, quantum capacitance, electrolyte gating, perfect absorber, graphene

## Abstract

Biosensors have emerged as a rapidly evolving area of research, offering transformative potential across biomedical diagnostics, environmental monitoring, and pharmaceutical applications. Among the diverse range of biosensing technologies, graphene-based surface plasmonic resonance (SPR) biosensors have attracted particular interest due to their exceptional sensitivity, scalability for mass production, and cost-effective fabrication processes. This study explores the operational principles and current design methodologies of graphene-based SPR biosensors, with a special emphasis on the role of electrolyte gating and its impact on sensor performance. Furthermore, the influence of graphene’s quantum capacitance is investigated as a critical parameter for improving the accuracy and reliability of performance predictions in the proposed sensor configuration. Computational analysis of sensitivity and key performance metrics was conducted. Notably, key performance metrics of the sensor improved upon incorporating quantum capacitance effects into the simulation framework. At a β_2_-microglobulin concentration of 0.00118 g/L, the sensitivity increased to 174 GHz·g/L, the figure of merit reached 0.55 L/g, the quality factor was 0.01, the signal-to-noise ratio (SNR) rose to 0.008, and the detection accuracy (DA) reached 0.08 L/THz, demonstrating the significant impact of quantum capacitance on the sensor’s performance. These findings highlight the potential of quantum-electrostatic considerations to enhance the precision and efficacy of graphene-based SPR biosensors, paving the way for the development of next-generation biosensing platforms with improved analytical capabilities. Unlike conventional graphene SPR biosensors, which primarily detect refractive index changes near the graphene surface, our model explicitly considers the electrostatic effect of biomolecules on graphene’s Fermi energy. By modelling β2-microglobulin as a charged species, we compute the resulting electric double layer and incorporate quantum capacitance in series. This amplifies the charge-induced modulation of graphene’s optical conductivity, and, combined with a graphene perfect absorber design, leads to enhanced plasmonic resonance shifts. Consequently, our approach achieves higher sensitivity and more precise detection of biomolecular interactions compared to traditional simulations.

## 1. Introduction

Biosensor technologies have gained considerable attention due to their capability to support early disease diagnosis, advance precision medicine, and enable applications such as genetic analysis and gene progression. As stated by the International Union of Pure and Applied Chemistry (IUPAC), a biosensor is an analytical device that combines a biological recognition element—like enzymes or immunological components—with a transducer to transfer biochemical interactions into measurable electrical, thermal, or optical signals [1,2].

In recent years, optical biosensors utilizing surface plasmonic resonance (SPR) have gained significant attention due to their high sensitivity and versatility. One of the key advantages of SPR technology is its ability to perform real-time, label-free detection of biomolecular interactions with high efficiency [3]. This makes it an invaluable tool across various scientific and industrial domains. SPR-based biosensors are increasingly being applied in fields such as agriculture and food safety, where they contribute to the detection of contaminants and the monitoring of food quality [4,5]. In the medical sector, they play a crucial role in diagnostics by enabling the identification of disease biomarkers [6,7]. Additionally, SPR has proven useful in environmental monitoring by detecting pollutants and harmful substances, as well as in industrial processes for quality control and safety assurance [8,9].

The primary function of SPR biosensors is the detection and analysis of a wide range of biomolecules, including lipids [10], peptides [11], receptors [12], nucleic acids [13], proteins [14], and antibodies [15]. This broad detection capability has made SPR a valuable technique in both research and applied sciences. Moreover, beyond biosensing applications, SPR is also widely used for the characterization of thin films and material surfaces, providing insights into film thickness, refractive index, and molecular adsorption behavior. These features highlight the versatility and growing importance of SPR in modern analytical science [16,17,18,19].

The first commercially available surface plasmonic resonance (SPR) instrument was introduced in 1990 by Biacore AB, a Swedish company. Since then, numerous manufacturers have developed and released various SPR systems to meet growing demand across different research and industrial sectors. Typically, an SPR instrument consists of three main integrated components: (i) an optical system featuring a high-refractive-index prism, (ii) a sensor chip coated with a thin metallic layer optimized for plasmonic excitation, and (iii) a microfluidic flow channel that delivers the analyte or sample to the sensor surface for analysis. Surface plasmonics are collective oscillations of free electrons that travel along the interface between a metal and a dielectric, with the associated electromagnetic field reducing exponentially away from the interface on both sides [20].

In recent years, graphene has attracted significant attention as a promising material for enhancing the performance of several biosensing platforms. Structurally, graphene consists of a one-atom-thick, two-dimensional sheet of carbon atoms with *sp*^2^ hybridization arranged in a hexagonal lattice [21]. This material presents superior properties, including very high charge carrier mobility, a two-dimensional architecture, and zero band gap [22]. Due to these properties and its relatively low cost, graphene may be used in the fabrication of excellent sensors [23].

Despite the significant advantages of graphene in SPR biosensors, several limitations remain in existing studies, particularly the insufficient consideration of electrostatic effects and the frequent neglect of graphene’s quantum capacitance, which can lead to inaccuracies in performance prediction. In parallel, alternative SPR configurations have been explored to enhance sensor sensitivity, such as fiber-optic SPR sensors incorporating grating structures. For instance, a recently reported D-shaped fiber SPR sensor utilizing an Ag–α-Fe_2_O_3_ composite grating demonstrated notable sensitivity enhancement. These developments highlight the diversity of design strategies in SPR technology. In this context, the present work introduces a graphene-based SPR biosensor model that incorporates quantum capacitance effects within an electrolyte-gated framework, enabling more accurate analysis and improved prediction of key performance parameters [24].

Among the proteins of clinical interest, β_2_-microglobulin (β_2_M) represents an important biomarker in several pathological conditions. β_2_M is a small (≈11.8 kDa) non-glycosylated protein that forms the light chain of major histocompatibility complex class I (MHC-I) molecules present on the surface of nearly all nucleated cells [25]. It is continuously released into the bloodstream during normal cellular turnover and is primarily cleared through glomerular filtration followed by tubular reabsorption in the kidneys [26]. As a consequence, circulating β_2_M levels are strongly correlated with renal function. In healthy individuals, serum β_2_M concentrations typically range between 1 and 3 mg/L, whereas significantly elevated levels are observed in patients with renal impairment, inflammatory disorders, and hematological diseases [27].

In individuals undergoing long-term hemodialysis, β_2_M concentrations may increase dramatically, often reaching 20–60 mg/L or higher, which can lead to protein aggregation and the development of dialysis-related amyloidosis [28]. Moreover, β_2_M is widely used as a prognostic biomarker in diseases such as multiple myeloma and lymphoma, where its serum concentration correlates with disease stage and tumor burden [29]. Due to its strong clinical relevance and the need for sensitive and rapid detection methods, β_2_M represents a valuable target for biosensing technologies. To date, previous studies have examined the behavior of these amyloidogenic proteins in the presence of different surfaces [30,31].

The concentration range considered in this study was chosen to capture both clinically relevant β2-microglobulin levels and the full device response. Physiological concentrations (~0.7–1.8 mg/L) and pathological levels (up to 20–60 mg/L, i.e., ~0.001–0.06 g/L) are included within the lower part of the simulated range, ensuring clinical relevance, while the extended range (up to 1.2 g/L) allows analysis of sensitivity and saturation effects. In the model, concentrations are converted to molar units and then to charge density via ρ = Σᵢ zᵢcᵢ, which enters the Poisson and Nernst–Planck equations to determine the electrostatic behavior self-consistently. Additionally, adsorption at the sensing interface links bulk concentration to an effective surface charge density [32,33].

**Figure 1:**
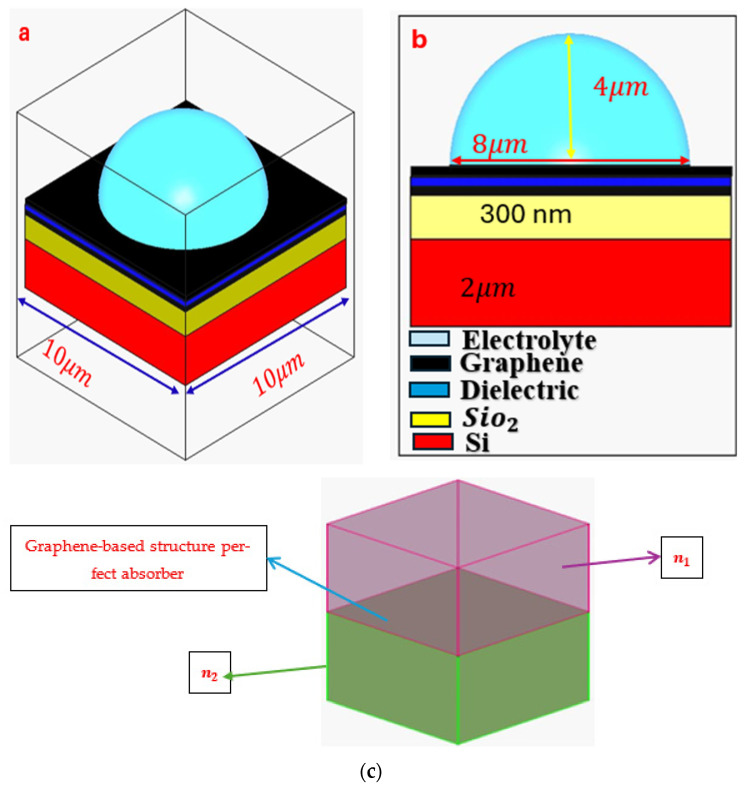
Schematic illustration of the surface plasmonic resonance (SPR) structure. (**a**) Three-dimensional view of the device. (**b**) Two-dimensional cross-sectional view. (**c**) Equivalent transmission-line model, where the conductive interface (e.g., a graphene layer) sandwiched between two dielectric media is represented as a load connected at the junction of two transmission lines.

In this study, a graphene-based perfect absorber sensor utilizing surface plasmonic resonance was simulated for the detection of β2-microglobulin protein. The simulation was conducted using COMSOL Multiphysics software Version 6.2. Figure 1 illustrates the structure of the proposed sensor along with its geometric dimensions.

## 2. Theory

Electrolyte-based electrostatic gating is a reliable method for adjusting the electronic response of atomically thin two-dimensional materials, such as graphene. Independent of the specific gate configuration, electrostatic doping functions through the same basic concept: using a voltage between graphene and the gate electrode induces charge accumulation, which alters the carrier concentration in graphene and hence shifts the Fermi level.

When a gate voltage is applied, an electrostatic potential difference (φ) is established between graphene and the gate electrode, leading to polarization at the graphene–electrolyte interface. Thus, ions and counterions rearrange to form an electrical double layer (EDL) on the electrolyte side of the interface. The formation of this EDL supports the accumulation of charge carriers with opposite polarity within the graphene layer, consequently causing a shift in its Fermi energy [34]. The gating pattern can be expressed by the following relationship [35]:

(1) $$E_{g}=\frac{E_{f}}{e}+\varphi$$

The Fermi energy in graphene changes as $E_{f}(n)={\hbar v}_{f}\sqrt{\pi n}$, where $v_{f}=1.1\times 10^{6}\frac{m}{s}$ is the Fermi velocity and n is the charge carrier density (doping concentration) induced by the top gate, with $\varphi =\frac{ne}{C_{EDL}}$. From Equation (1), we obtain and $E_{g}$ is the Fermi energy of graphene after applying electro gating [35].

(2) $$E_{g}=\frac{{\hbar v}_{f}\sqrt{\pi n}}{e}+\frac{ne}{C_{EDL}}$$

By using constant parameters, the final equation, which allows us to estimate the doping concentration at each top-gate voltage, becomes the following:

(3) $$E_{g}={1.16\times 10}^{-7}\sqrt{n}+\frac{ne}{C_{EDL}}$$

In this work, the electrolyte is modelled as an aqueous solution containing β2-microglobulin as the target analyte. The electrolyte forms an electrical double layer (EDL) at the interface, characterized by the capacitance $C_{EDL}$, which captures the ionic environment. Variations in analyte concentration modulate the carrier density n, thereby influencing the Fermi level and gating potential as described in Equations (1)–(3) [36].

The first and simplest model introducing the concept of the formation of a double layer at the electrochemical interface and thus describing the structure of the EDL was formulated by Helmholtz. Helmholtz envisaged that the total charge on the electrode is balanced by a monolayer of ions of opposite charge located adjacent to the surface, referred to as the Helmholtz layer. Therefore, the Helmholtz layer capacitance is computed through the below equation [37,38,39,40,41,42]:

(4) $$C_{H}=\frac{\varepsilon_{0}\varepsilon_{H}}{d_{H}}$$

where $\varepsilon_{0}$ is the permittivity of free space, $\varepsilon_{H}$ is the relative permittivity of the electrolyte inside the Helmholtz layer, and $d_{H}$ is the size of the solvated ions [39].

Another theoretical framework used to describe the electrical double layer is the Gouy–Chapman model. In this framework, a theoretical expression for the capacitance per unit area of the electrical double layer is derived as follows [7,38,41]:

(5) $$C_{Gc}=\frac{\varepsilon_{r}\varepsilon_{0}}{\lambda_{D}}cosh(\frac{2e\varphi }{2K_{B}T})$$

where $\lambda_{D}$ is a Debye length, which is given by $\lambda_{D}=({\frac{2ce^{2}}{\varepsilon_{r}\varepsilon_{0}K_{B}T})}^{-\frac{1}{2}}$ for a monovalent electrolyte, where c is the concentration of the electrolyte, e is the electric charge, and $K_{B}T$ is the thermal energy [42].

An additional advancement in formulating a complete description of the electrical double layer (EDL) was introduced by Stern, who proposed integrating the Helmholtz and Gouy–Chapman models. As a result of this combined approach, the EDL can be considered as two electrical capacitors connected in series. Consequently, the inverse total EDL capacitance can be written as follows [43]:

(6) $$\frac{1}{C_{EDL}}=\frac{1}{C_{H}}+\frac{1}{C_{Gc}}$$

While extensive research on graphene has emphasized its exceptional electrical and structural characteristics, an equally important parameter—quantum capacitance—has received comparatively limited attention. In the present study, direct measurements of the quantum capacitance of graphene were performed under varying protein concentrations to explore its sensitivity to biomolecular interactions. The results show that the quantum capacitance exhibits a non-zero minimum at the Dirac point. These findings align with prior studies, including those by Jilian Xia et al., which suggest that charged impurities in the graphene environment play a significant role in modulating quantum capacitance by altering the local carrier density. Such deviations from ideal theoretical behavior underscore the impact of disorder and environmental factors on graphene’s electronic response [44].

To carry out the theoretical computation, a minimal model was employed in which the impurity concentration serves as the sole adjustable parameter. For ideal, impurity-free graphene, the quantum capacitance can be analytically derived based on the density of states arising from its linear energy–momentum dispersion relation [44]:

(7) $$C_{Q}=\frac{2e^{2}K_{B}T}{\pi ({\hbar v}_{f}{)}^{2}}Ln[2\left(1+cosh\frac{eV_{ch}}{K_{B}T}\right)]$$

where ħ is the Planck constant, e is the electron charge, $K_{B}$ is the Boltzmann constant, $v_{f}=\frac{C}{300}$ is the Fermi velocity of the Dirac electron (the symbol *c* represents the speed of light in a vacuum), and $V_{ch}=\frac{E_{f}}{e}$ is the potential of graphene. When $eV_{ch}\gg K_{B}T$, Equation number (7) is reduced to the below Equation (8):

(8) $$C_{Q}=\frac{2e^{2}}{{\hbar v}_{f}\sqrt{\pi }}\sqrt{n}$$

The quantum capacitance of graphene is directly related to its charge carrier density, as shown in Equation (8). In realistic conditions, charged impurities or adsorbed molecules modify the local carrier density, leading to a finite density of states near the Dirac point and thus a non-zero minimum quantum capacitance [36]. In this work, β2-microglobulin molecules (charge −2) are treated as impurities that alter the graphene charge density, and the impurity concentration used in the model is consistent with typical experimental graphene-based sensing systems.

The total capacitance of a graphene-based system in an electrolyte environment arises from the series combination of two distinct components: the quantum capacitance of graphene and the electric double-layer (EDL) capacitance formed at the graphene–electrolyte interface. Due to this series configuration, the overall capacitance $C_{t}$ is governed by the reciprocal sum of the individual capacitances, expressed as follows [45,46]:

(9) $$\frac{1}{C_{t}}=\frac{1}{C_{EDL}}+\frac{1}{C_{Q}}$$

**Figure 2:**
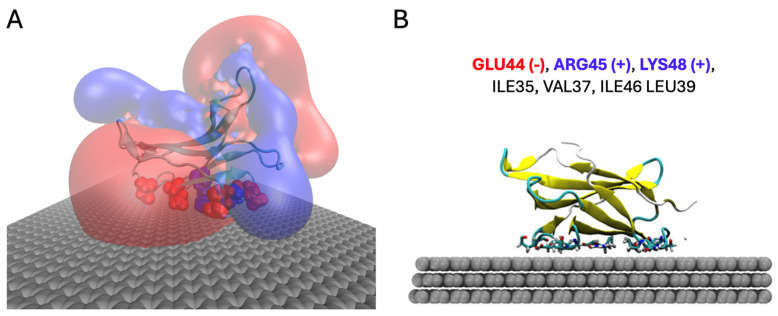
(**A**) Electrostatic potential map of β_2_M adsorbed on graphene, obtained from molecular dynamics (MD) simulations. The results highlight that the electric double layer (EDL) near the graphene surface is influenced not only by small electrolyte ions but also by the presence of large biomolecules. Positively charged residues of the protein are shown in blue and negatively charged residues in red, illustrating their spatial contribution to local electrostatic potential modulation at the interface. (**B**) Final confirmation of β_2_M adsorbed on graphene after 3.1 μs of Temperature Replica Exchange Molecular Dynamics (T-REMD) simulation, showing the arrangement of charged and neutral residues in contact with the graphene surface.

In this research, we use electrolytes containing $\beta_{2}$-microglobulin (β_2_M) at different concentrations as a sample to affect the charge density of graphene; consequently, the Fermi energy of graphene will be changed. Results of the molecular dynamics (MD) simulations conducted in the PhD thesis of Maschio [47] showed that the EDL near the graphene surface is affected not only by ions but also by large biomolecules like β_2_M, since, upon approaching the graphene surface, β_2_M adsorbs and adopts a preferred orientation in which specific amino acid residues interact directly with the substrate, as illustrated in Figure 2A. Because β_2_M has a distinct size, shape, and surface charge distribution, it influences the local potential differently than small ions. β_2_M is a protein of 12 kDa in terms of molecular mass and it has a diameter of around 3–4 nanometers. When β_2_M molecules are present near the graphene surface (e.g., in a solution at 50 mM), they can alter the Helmholtz potential in a manner specific to its total charge and spatial arrangement. More specifically, results of extensive molecular dynamics simulations, with 3.1 µs Temperature Replica Exchange Molecular Dynamics (T-REMD) based on the OPLS-AA force field with the SPC/E explicit water model as implemented in the GROMACS package [48], revealed that the protein tends to lie horizontally on the graphene surface with a patch that involves the C strand (Ile35, Val37, Leu39), C’ strand (Glu44, Arg45), and CD loop (Ile46, Lys48). Among these residues, Glu44 is a negatively charged, Arg45 and Lys48 are positively charged, and Ile35, Val37, Leu39, and Ile46 are neutral, as in Figure 2B.

Since β_2_M can adsorb directly, it will generate a stable and distinct signal due to its size and charge. This adsorption alters the graphene’s surface potential, detectable by changes in the sensor response. In this work, we adopt a simplified model in which the protein is assigned a net charge of −2, based on the total protonation state calculated at neutral pH using H++ 1.0 software. Additionally, the effective protein diameter of 4 nm was obtained from atomistic simulations and included as an input parameter in the COMSOL calculations, as well as the protein self-diffusion coefficient. In future work, we aim to investigate the effects of varying ionic strength.

Graphene shows a series of outstanding features that make it highly attractive for photonic and optoelectronic applications. Nevertheless, the inherently weak optical absorption of monolayer graphene greatly hinders its practical use. To overcome this limitation and substantially enhance light–matter interaction, a diversity of graphene-based architectures has been suggested to realize near-perfect absorption of incident electromagnetic waves [49].

In the mid-infrared (IR) to terahertz (THz) domain, graphene supports plasmonic resonances that dramatically boost its near-field. Plasmons are collective oscillations of charge carriers [50], and in doped graphene, they can be tuned via electrostatic gating or chemical doping. In comparison with metals, graphene plasmons have longer lifetimes, lower losses, and much larger wave vectors, resulting in highly confined electromagnetic fields. These properties enable enhanced optical absorption and even near-perfect absorption of incident light [51].

The effective surface conductivity method employs a transmission line analogy. Propagation of a plane wave in a dielectric can be described as a transmission line with ideal electric and magnetic boundaries. A thin conducting layer with complex surface conductivity $\sigma_{s}=\sigma_{s}^{\prime }+i\sigma_{s}^{\prime \prime }$ at the interface of two dielectrics (with refractive indices $n_{1}$ and $n_{2}$, see Figure 1c) can be expressed as a load connected between the two transmission lines [52].

When the conductive layer’s thickness is much smaller than the wavelength, it can be approximated as a point load. For normal incidence, the amplitude transmission and reflection coefficients for a wave incident from the first dielectric are given by the following:

(10) $$r=\frac{n_{1}-n_{2}-\sigma_{s}Z_{0}}{n_{1}+n_{2}+\sigma_{s}Z_{0}}$$

(11) $$t=\frac{2n_{1}}{n_{1}+n_{2}+\sigma_{s}Z_{0}}$$

$Z_{0}=120\pi$ Ohm is the free-space impedance [53].

In our modelling, graphene is considered as a conductive surface, which significantly reduces the run time. The absorption rate is determined by the equation $A\left(\omega \right)=1-R\left(\omega \right)-T(\omega )$, where $R\left(\omega \right)$ and T(ω) represent the reflectance and transmittance, respectively. Due to the complete absence of transmission through the perfect electric conductor layer, the absorption relation is simplified as $A\left(\omega \right)=1-R\left(\omega \right)$ [54].

Although water exhibits strong absorption in the THz frequency range, THz interactions with aqueous electrolytes can still be analyzed through their dielectric response over short propagation distances. In this work, the dielectric properties of the electrolyte solution are described using the Debye–Falkenhagen theory of ionic atmosphere relaxation, which accounts for the frequency-dependent dielectric behavior of electrolyte solutions. According to this theory, the dielectric constant of the solution increases with the square root of the ion concentration and can be expressed as $\varepsilon_{sol}=\varepsilon_{H_{2}O}+A\sqrt{c}$, where $\varepsilon_{H_{2}O}$ is the dielectric constant of pure water, c is the electrolyte concentration, and A is a coefficient dependent on the electrolyte type [55].

## 3. Effective Conductivity of Graphene Metamaterials

In addition to its linear energy dispersion, graphene’s optical conductivity is fundamental to its optical and plasmonic properties, as it determines how graphene interacts with external signals such as electromagnetic waves or fast-moving electrons. The conductivity usually includes two components: intraband transitions, which occur within the same energy band and do not conserve momentum, and interband transitions, which involve electrons moving from the valence to the conduction band while conserving momentum, namely

(12) $$\sigma_{gr}=\sigma_{intra}(\omega )+\sigma_{inter}(\omega )$$

It can be derived with the random phase approximation (RPA) theory:

(13) $$\sigma_{intra}\left(\omega \right)=\frac{2iK_{B}Te^{2}}{\pi \hbar^{2}(\omega +i\tau^{-1})}Ln(2cosh(\frac{E_{f}}{2K_{B}T}))$$

(14) $$\sigma_{inter}\left(\omega \right)=\frac{e^{2}}{4\hbar }[\frac{1}{2}+\frac{1}{\pi }+\arctan\left(\frac{\hbar \omega -E_{f}}{2K_{B}T}\right)-\frac{i}{2\pi }Ln\frac{{\left(\hbar \omega +E_{f}\right)}^{2}}{({\hbar \omega -E_{f})}^{2}+{\left(2K_{B}T\right)}^{2}}]$$

where e is the electron charge, $K_{B}$ is the Boltzmann constant, ħ is the reduced Planck’s constant, T is the temperature, and ω is the angular frequency of incident light. τ is the relaxation time and $E_{f}$ is the Fermi level [56,57,58,59].

Although the structure investigated in this study has been simulated numerically, all of its components are realizable using conventional experimental fabrication techniques as described in [60,61]. High-quality graphene, grown through chemical vapor deposition (CVD), has been widely produced in laboratories and can be transferred onto various dielectric substrates, including transparent and polar substrates, using wet transfer methods. Metallic layers, such as gold, can be readily patterned and deposited as nanostructures with precise dimensions on different substrates via electron beam lithography and thermal evaporation. The interaction between metal and graphene can be controlled by incorporating thin dielectric spacer layers, such as Al_2_O_3_ or h-BN, with thicknesses of a few nanometers. These spacer layers are commonly employed in nanophotonics laboratories using techniques like atomic layer deposition (ALD) or dry transfer methods.

In our device, the Fermi energy of graphene is modulated via electrostatic gating induced by protein adsorption near the graphene surface, without applying an external gate voltage. This mechanism enables real-time, label-free detection. A major challenge is the orientation of β2-microglobulin (B2M) upon adsorption, which significantly influences the Fermi level shift and the SPR response. Surface functionalization and buffer optimization are employed to minimize nonspecific adsorption and improve reproducibility.

## 4. Results and Discussion

To calculate the electric field and electrostatic potential within the Electric Double Layer (EDL) caused by the presence of a protein near the graphene surface, we begin with the Poisson equation. After designing the computational model, we generated a series of plots and graphs to visualize and compare key parameters. These visualizations allowed for a comprehensive evaluation of how incorporating quantum capacitance influences both the electric field and potential. The electric field distribution and mass transport pattern were analyzed by solving an electrostatic problem governed by the Poisson equation, $\nabla \left(\varepsilon \varepsilon_{0}\nabla \varphi \right)=0$, that was solved in the compact layer. In the diffuse layer, a coupled Nernst–Planck and electrostatic model was employed to capture the transport phenomena more accurately [62]

(15) $$\frac{{\partial c}_{i}}{\partial t}=\nabla (D_{i}\nabla c_{i}+z_{i}FD_{i}c_{i}\nabla \frac{\varphi }{RT})$$

and $\nabla \left(\varepsilon \varepsilon_{0}\nabla \varphi \right)=\rho$, where $\rho =\sum z_{i}c_{i}$, is solved in the electrolytic domain outside of the compact layer. Here, $D_{i}$, $z_{i}$, and $c_{i}$ are respectively the diffusivity, the charge valence, and the concentration of the protein species i, t is time, F is the Faraday constant, R is the gas constant, T is the absolute temperature, φ is potential, ε is the dielectric constant, $\varepsilon_{0}$ is the vacuum permittivity, and ρ is the charge density. The coupled Poisson–Nernst–Planck equations (Equation (15)) were solved in the electrolyte domain outside the compact layer, modeled as a spherical droplet of radius 4 nm in contact with the graphene surface. At the graphene–electrolyte interface, a fixed surface potential (or equivalently surface charge) boundary condition was applied to account for electric double layer formation, while the electric potential was set to zero at the outer droplet boundary to represent bulk electroneutral conditions. β2M was treated as a mobile ionic species with valence z = −2 (based on molecular dynamics results), and its transport was described using the self-diffusion coefficient for the protein calculated with HYDROPRO and included in the calculation as 0.0135 Å^2^/ps at T = 300 K and pH = 7.

**Figure 3:**
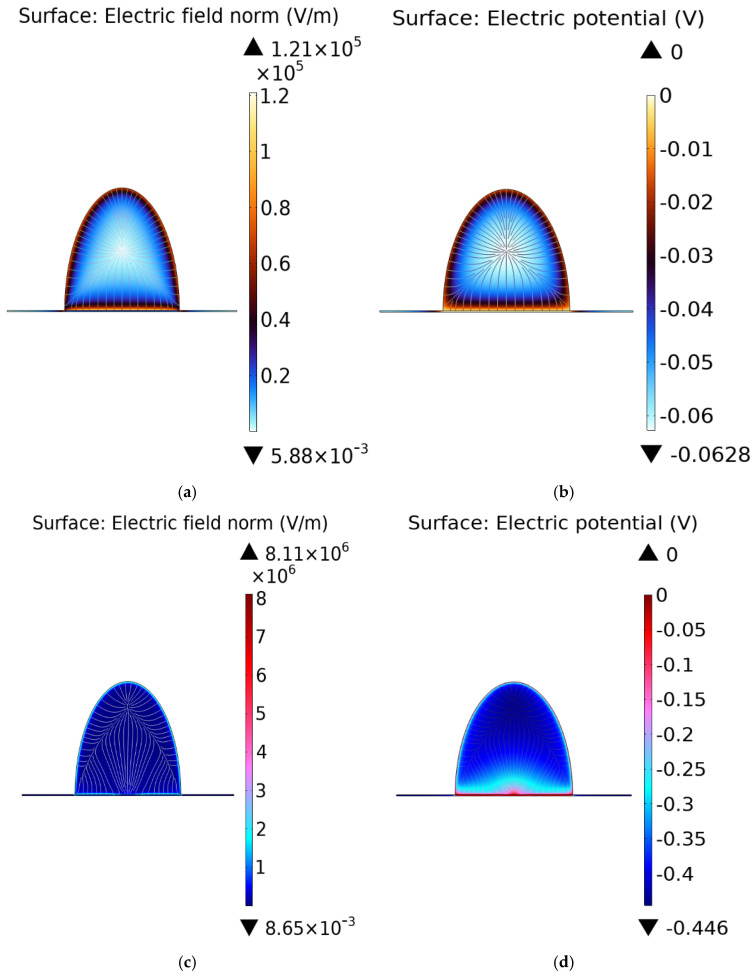
Electric double layer (EDL) models and their electrical responses. (**a**) Electric field distribution and (**b**) electric potential profile in the conventional Stern model of the EDL. (**c**) Electric field distribution and (**d**) electric potential profile for the EDL when the quantum capacitance of graphene is included, showing the modified electrostatic response compared to the classical Stern model.

Figure 3a–d illustrates the distribution of electric potential and electric field within the electric double layer (EDL).

**Figure 4:**
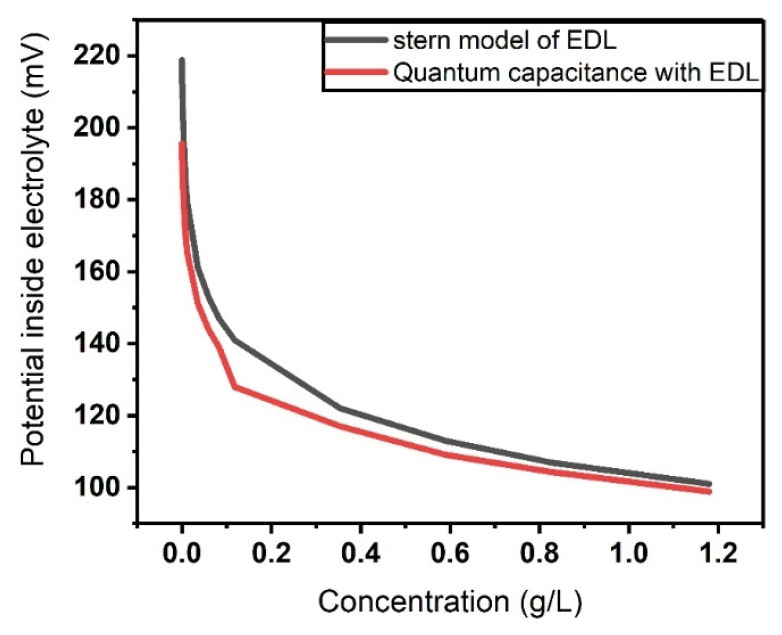
Electrolyte potential for (black color) the Stern model of the EDL and (red color) the Stern model of the EDL considering the effect of quantum capacitance.

The next step is calculation of the potential within the layer at different concentrations of $\beta_{2}-microglobulien$ and varying distances from the electrolyte–graphene interface. Due to the pivotal influence of the electrolyte potential within the broader computational model, this parameter was first assessed at two distinct concentrations while systematically altering the separation from the electrode–electrolyte boundary. The outcomes of these simulations are illustrated in Figure 4 and align well with the trends predicted using Equation (16) [43].

(16) $$\varphi \left(x\right)=\varphi_{0}\left(x\right)exp(\frac{-x}{\lambda_{D}})$$

The term $\phi_{0}$ represents the effective surface potential at the electrolyte–graphene interface (x = 0), which serves as the boundary condition for the exponential decay of the electrolyte potential and depends on the local β_2_-microglobulin concentration through its influence on the interfacial charge density.

**Figure 5:**
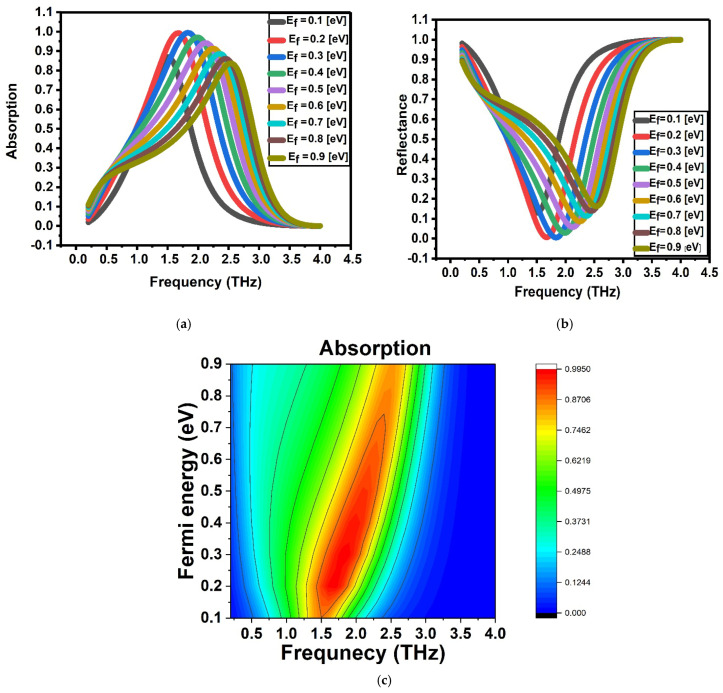
Optical response of graphene at different Fermi energy levels. (**a**) Absorption spectra and (**b**) reflectance spectra of graphene for Fermi energy values ranging from 0.1 to 0.9 eV (in steps of 0.1 eV), illustrating the tunability of graphene’s optical properties through Fermi level modulation. (**c**) Colormap of absorption as a function of photon energy and Fermi energy, providing a comprehensive visualization of the absorption dependence on Fermi level.

To investigate the broadband absorption properties of the graphene-based structure, a frequency-domain solver utilizing the finite element method (FEM) is adopted. In order to suppress transmission and maximize electromagnetic wave absorption within the infrared to terahertz frequency range, a perfect electric conductor (PEC) is strategically placed as a back reflector. This setup serves to eliminate transmitted waves while simultaneously minimizing reflection, thereby enabling highly efficient light absorption across the targeted spectrum. Figure 5 illustrates the band spectrum of the proposed structure. Since the primary objective of this study is to investigate the influence of electrostatic gating on the absorption and reflection performance—specifically, the effect of varying the Fermi energy of graphene—we evaluate the absorption and reflection characteristics at different Fermi energy levels. After identifying the Fermi energy value at which maximum absorption and minimum reflection occur, that optimal value is selected for further analysis and implementation.

In Figure 5, the Fermi energy of graphene was varied parametrically in the range of 0.1–0.9 eV to evaluate its influence on the optical response and determine the optimal operating point. At this stage, the electrostatic results (potential and concentration distributions) were not directly coupled to the optical model; the figure represents an independent parametric study. The electrostatic–optical coupling mechanism is described in the subsequent section.

The graphene-based structure exhibits its highest absorption performance at a Fermi energy level of 0.3 eV. At this specific value, the absorption reaches approximately 99.211%**,** while the corresponding reflection is minimized to an exceptionally low value of 0.00789. These results indicate that the structure is capable of near-perfect absorption and negligible reflection under the given conditions. Owing to this optimal performance, all subsequent simulations and analyses are carried out using a Fermi energy of 0.3 eV, as it ensures the most efficient interaction between the incident electromagnetic waves and the graphene layer.

In the following section, we investigate the effect of specific concentrations of β2-microglobulin on the variation of the Fermi energy and the quantum capacitance of graphene. As previously discussed, the presence of this protein in proximity to the graphene surface induces a modification in the local charge carrier density. This alteration occurs due to the interaction between the protein’s electric field and the delocalized π-electrons of graphene. Since both Fermi energy and quantum capacitance are directly dependent on the charge carrier density, as described by Equations (2) and (8), any variation in the protein concentration will consequently lead to measurable changes in these parameters.

**Figure 6:**
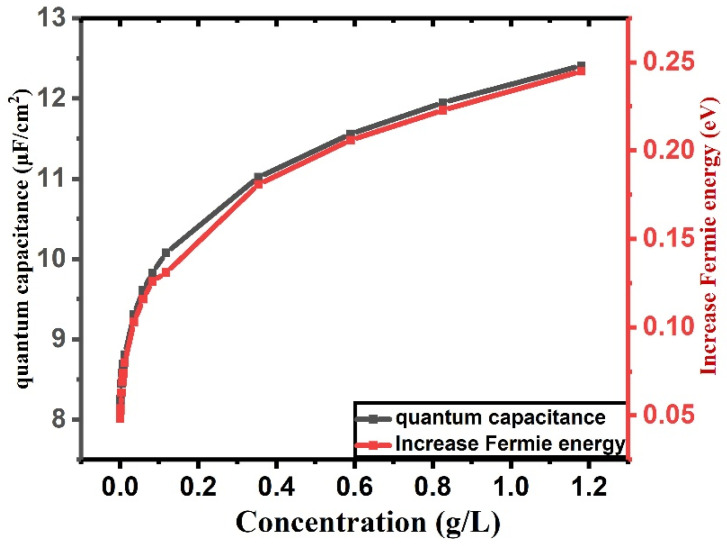
Variation of quantum capacitance and corresponding Fermi energy shift as a function of β_2_-microglobulin concentration. The figure illustrates how changes in biomolecule concentration modulate the electronic properties of graphene, reflected by variations in quantum capacitance and shifts in the Fermi energy.

Figure 6 illustrates the quantitative effects of different concentrations of β2-microglobulin on the Fermi energy and quantum capacitance. As the concentration increases, the perturbation to the charge distribution becomes more significant, resulting in a noticeable shift in the Fermi level. This shift, in turn, affects the quantum capacitance, which is sensitive to changes in the electronic density of states near the Dirac point.

In this study, the primary objective is to investigate the effect of protein concentration on the optical properties of graphene-based perfect absorber structure, specifically focusing on the shift in the absorption peak position and the minimum reflectance wavelength. The analysis explores how varying concentrations of β2-microglobulin protein near the graphene surface influence its electronic properties, particularly the Fermi energy level.

As the concentration of β2-microglobulin increases, the extent of Fermi level alteration becomes more pronounced, resulting in a larger shift in the resonance frequency observed in the absorption spectrum. Notably, this shift is directed towards higher frequencies, corresponding to a rightward shift in the spectral position. This rightward shift can be attributed to the interaction between the protein molecules and graphene’s surface, which effectively dopes the graphene and increases its carrier concentration. This doping modifies the plasmonic behavior of graphene, causing the resonance peaks to move to shorter wavelengths (higher energies).

**Figure 7:**
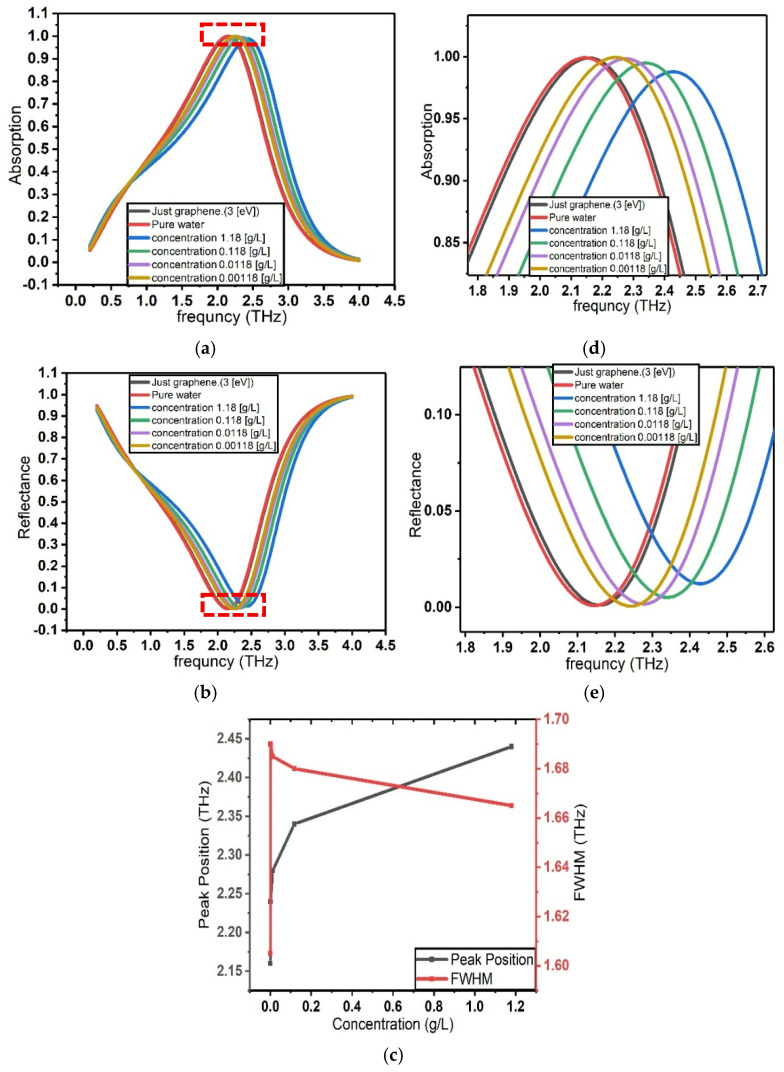
Optical response of graphene in the presence of different concentrations of β_2_-microglobulin within the Stern model of the electrical double layer (EDL). (**a**) Absorption spectra of graphene for varying β_2_-microglobulin concentrations. (**b**) Enlarged view of the absorption spectra highlighting the shift in the resonance peak. (**c**) Reflectance spectra of graphene for different β_2_-microglobulin concentrations. (**d**) Enlarged view of the reflectance spectra showing the resonance peak shift. (**e**) Corresponding resonance peak position and full width at half maximum (FWHM) as a function of analyte concentration, demonstrating the concentration-dependent optical response.

To accurately model the interaction between charged biomolecules and the graphene surface, the Stern model for the electrical double layer is employed. Figure 7 illustrates this shift clearly, showing the progressive movement of the absorption peak and reflectance minimum to the right as the β2-microglobulin concentration increases. This visualization confirms the direct correlation between protein concentration and the spectral shift of graphene’s optical features.

For this research, physiologically relevant concentrations of β2-microglobulin protein are selected: 0.00118 g/L, 0.0118 g/L, 0.118 g/L, and 1.18 g/L. These concentrations correspond to levels commonly found in biological systems and are therefore particularly significant for medical diagnostics and biosensing applications.

**Figure 8:**
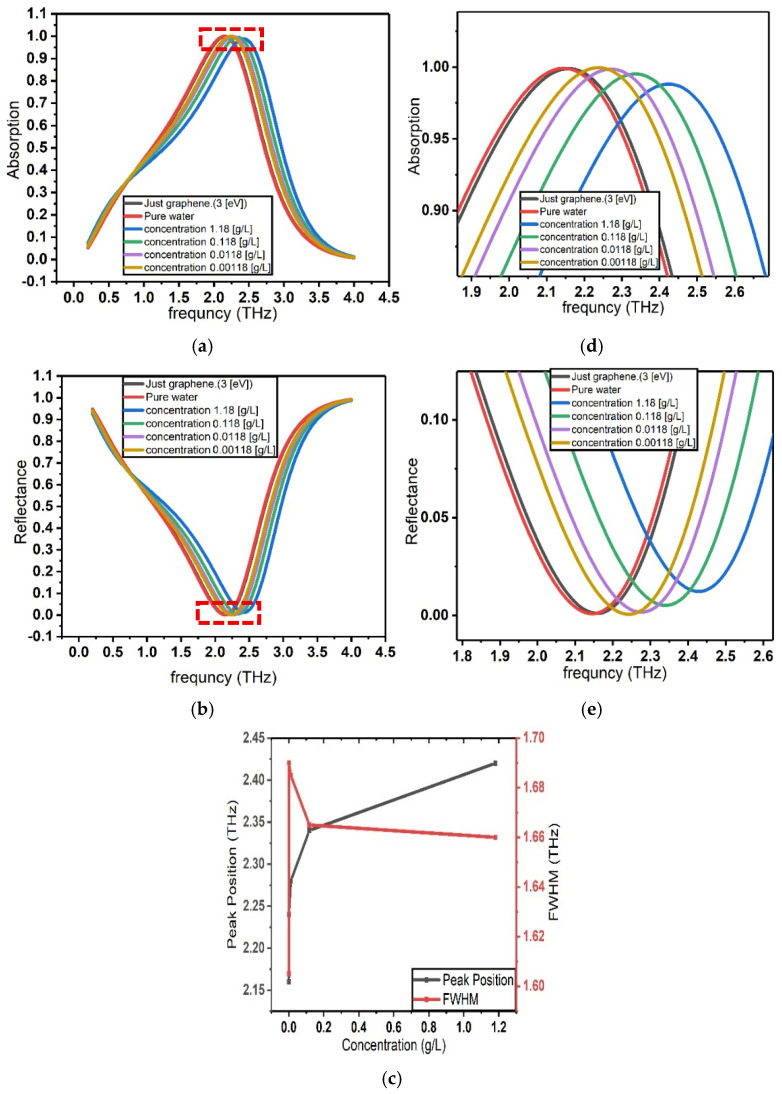
Optical response of graphene in the presence of different concentrations of β_2_-microglobulin within the Stern model of the electrical double layer (EDL), including the effect of quantum capacitance. (**a**) Absorption spectra of graphene for varying β_2_-microglobulin concentrations. (**b**) Reflectance spectra of graphene for different β_2_-microglobulin concentrations. (**c**) Corresponding resonance peak position and full width at half maximum (FWHM) as a function of analyte concentration, illustrating the impact of quantum capacitance on the concentration-dependent optical response. (**d**) Enlarged view of the absorption spectra highlighting the shift in the resonance peak (region indicated by the red dashed box). (**e**) Enlarged view of the reflectance spectra showing the corresponding resonance peak shift.

To obtain more accurate results that align closely with experimental observations, it is essential to account for all relevant factors influencing the computational model. Therefore, in Figure 8, the same optical parameters of the graphene-based perfect absorber are calculated while incorporating the impact of quantum capacitance. Considering quantum capacitance allows for a more realistic representation of the charge accumulation behavior in graphene, particularly under conditions where the density of states near the Dirac point significantly affects the system’s electrostatic and optical response. The strong influence of quantum capacitance arises from the low density of states of graphene near the Dirac point, where its quantum capacitance (C_Q) becomes comparable to the electric double layer capacitance (C_EDL). Since the total interfacial capacitance is determined by their series combination, inclusion of C_Q modifies the charge–potential relationship and leads to a larger modulation of carrier density and Fermi level. Because the optical conductivity of graphene is directly governed by the Fermi energy, this enhanced Fermi-level shift results in a stronger optical response and improved sensor sensitivity.

The quantum capacitance of graphene is incorporated into the Multiphysics model through its electrostatic coupling with the electrical double layer (EDL) formed at the graphene–electrolyte interface. The electrochemical potential of graphene is determined by the Fermi energy and the interfacial potential according to $E_{g}=E_{F}/e+\phi$, where $E_{F}$ is the Fermi energy, e is the elementary charge, and ϕ is the electrostatic potential at the interface. The potential drop across the electrolyte is related to the surface charge density n through the EDL capacitance as $\phi =ne/C_{EDL}$. To account for the finite density of states in graphene, the quantum capacitance $C_{Q}$ is included in series with the EDL capacitance, resulting in a total interfacial capacitance given by $1/C_{tot}=1/C_{EDL}+1/C_{Q}$. This series capacitance modifies the potential distribution at the graphene–electrolyte interface and consequently determines the charge density and Fermi energy of graphene within the Multiphysics simulation.

## 5. Performance Evaluation Metrics

Sensor functionality is affected by multiple variables, making it essential to consider these factors when assessing overall performance. The accuracy and reliability of sensor measurements can be impacted by a range of influences. Key performance metrics used to evaluate sensor effectiveness are outlined below [63,64]:

(17) $$S=\frac{∆f}{∆C}$$

(18) $$FOM=\frac{S}{FWHM}$$

(19) $$Q=\frac{f_{r}}{FWHM}$$

(20) $$SNR=\frac{∆f}{FWHM}$$

(21) $$DA=\frac{1}{FWHM}$$

(22) $$\mathrm{LOD}=\frac{3\;\sigma_{b}}{S}$$

In this context, *f* denotes the frequency, *c* represents the protein concentration, $f_{r}$ is the peak position of absorption, *S* indicates the sensitivity, *FWHM* refers to the full width at half maximum, *Q* is the quality factor, *FOM* stands for the figure of merit, *SNR* denotes the signal-to-noise ratio, and *DA* represents detection accuracy. Table 1 illustrates the sensor’s performance at two protein concentrations, representing the highest and lowest concentrations used in this study. LOD is the limit of detection, where $\sigma_{b}$ is the standard deviation of the baseline signal measured at zero analyte concentration (c=0), and S is the sensitivity, defined as the slope of the calibration curve relating the resonance peak shift in the THz absorption spectrum to the analyte concentration.

A comparison with previously reported SPR sensors shows that the proposed design offers improved performance. The obtained sensitivity (84.74 THz × g/L) is higher than that reported in [63] (80.71 THz × g/L) and [64] (72.05 THz*g/L), demonstrating the enhanced sensing capability of the proposed structure for protein detection

## 6. Conclusions

In conclusion, we have presented a novel design for a perfect absorber biosensor operating in the terahertz frequency range. The proposed structure features a layered configuration of graphene/dielectric/graphene, which enables dynamic tunability by adjusting the Fermi energy of the graphene layers. Numerical simulations demonstrate that, by varying the doping level from 0.1 eV to 0.9 eV, the absorber exhibits distinct absorption peaks, with notable resonance observed around 0.3 eV in the terahertz range.

To explore biosensing applications, we introduced an electrolyte containing the protein β2-microglobulin to polarize the graphene surface and modulate its Fermi level. As the concentration of the protein increases, a corresponding shift in the absorption peak is observed, confirming the sensor’s responsiveness to biochemical changes. The sensitivity of the biosensor was calculated to be 220 GHz·L/g at the maximum concentration (1.18 g/L) and 67,796 GHz·L/g at the minimum concentration (0.00118 g/L).

Furthermore, we evaluated the impact of the quantum capacitance of graphene on the sensor’s performance. The results indicate that accounting for quantum capacitance—beyond the conventional electrical double-layer model—significantly enhances sensitivity. Under the same concentration range, the improved sensitivities were 589.5 GHz·L/g and 67,970 GHz·L/g, respectively. This innovative absorber structure demonstrates strong potential for the development of tunable terahertz biosensors based on graphene–dielectric–graphene composites, offering high sensitivity and dynamic control for advanced biochemical detection applications.

Future work will focus on experimental validation of the computational model by fabricating graphene-based SPR sensors, controlling protein adsorption and orientation, and performing real-time SPR measurements in biological fluids to assess sensor performance and reproducibility.

## Figures and Tables

**Table 1:** Key parameters influencing the performance of a graphene-based sensor under different electrical double layer (EDL) models at two concentrations of β_2_-microglobulin. The table compares key performance metrics—sensitivity (S), figure of merit (FOM), quality factor (Q), signal-to-noise ratio (SNR), detection accuracy (DA), and limit of detection (LOD)—highlighting the effects of EDL modelling and biomolecule concentration on overall sensor performance.

Sensor Parameter Performance	C = 1.18 [g/L]		C = 0.00118 [g/L]	
	EDL in Stern Model	EDL + Quantum Capacitance	EDL in Stern Model	EDL + Quantum Capacitance
S (THz L/g)	0.169	0.254	33.9	84.74
FOM (L/g)	0.097	0.141	20.42	51
Q	1.38	1.39	1.35	1.38
SNR	0.115	0.166	0.023	0.06
DA (1/THz)	0.58	0.55	0.574	0.602
LOD (g/L)	0.017	0.018	8.8 × 10^−5^	3.5 × 10^−5^

## Data Availability

Data is contained within the article or Supplementary Materials.

## Supplementary Materials

The following supporting information can be downloaded at https://www.mdpi.com/article/10.3390/s26092815/s1. Figure S1. Non-uniform mesh distribution used in the simulations. A highly refined mesh is applied in regions with strong field variation, particularly near the graphene layer and the electric double layer (EDL), with gradually reduced density elsewhere. A convergence study confirms negligible variation beyond a certain mesh density. Table S1. Complete set of simulation parameters, including material properties, geometrical dimensions, and physical constants.
