# Supplementary material for: Computational Simulation of a Surface Plasmonic Resonance Biosensor for β2-Microglobulin Based on Electrolyte-Gated Graphene
Source: Sensors (Basel). 2026 Apr 30;26(9):2815. doi: 10.3390/s26092815 (PMC13165553; doi:10.3390/s26092815)
Supplement: Supplementary file 1 [file sensors-26-02815-s001.zip › sensors-4172799-supplementary.pdf]

Computational Simulation of Surface Plasmon Resonance Biosensor for β2-Microglobulin based on Electrolyte-Gated Graphene

Ghassem Baridi [1,*], Arslan Liaquat [2], Leonardo Martini [2], Federico Rapuzzi [2], Vito Clericò [3], Mario Amado[3],
Enrique Diez[3], El Hadj Abidi [3], Maria Celeste Maschio [2], Stefano Corni [4], Yahya Moubarak Meziani [3],
Giorgia Brancolini [4], Francesco Rossella [2] and Luigi Rovati [1]

[1] Department of Engineering "Enzo Ferrari", University of Modena and Reggio Emilia, Via P. Vivarelli, 10, 41125 Modena, Italy

[2] Department of Physics, Computer Science and Mathematics, University of Modena e Reggio Emilia, Via Campi 213/a, 41125 Modena, Italy

[3] Department of Applied Physics, University of Salamanca, 37008 Salamanca, Spain

[d] Nanoscience Institute-Esse 3, National Research Council, Via Campi 213/a, 41125 Modena, Italy

Boundary Conditions:

The graphene layer is modelled using a transition boundary condition, which allows it to be treated as an infinitesimally thin conductive sheet with equivalent surface conductivity, effectively behaving as a transmission line. The top surface of the structure is defined as a port boundary to excite the system. The remaining boundaries are assigned perfect electric conductor *(PEC)* and perfect magnetic conductor *(PMC)* conditions to ensure proper field confinement and eliminate spurious reflections.

Electrolyte Modelling:

The electrolyte is modelled under physiological conditions (pH = 7, room temperature). The electric potential is assumed to decay exponentially away from the graphene-electrolyte interface, approaching zero at the bulk electrolyte boundary. The relative permittivity of the electrolyte is taken as 78.5, corresponding to water.

Debye Length Calculation:

The Debye length (λ_D) is calculated using:

$$\lambda_D = \left(\frac{2ce^2}{\varepsilon_r \varepsilon_0 k_B T}\right)^{-1/2}$$

where c is the ion concentration, e is the elementary charge, ε_0 is the vacuum permittivity, k_B is Boltzmann's constant, and T is the temperature. For the Stern layer, a relative permittivity of ε_r= 11.5 is used. The Debye length is evaluated for different electrolyte concentrations and incorporated into the simulation domain.

Mesh Convergence Analysis:

A non-uniform meshing strategy is employed. A highly refined mesh is applied in regions with strong field variation, particularly near the graphene layer and the electric double layer (EDL). The mesh density is gradually reduced away from these regions to optimize computational efficiency. A convergence study was performed by comparing results obtained with progressively finer meshes; negligible variation was observed beyond a certain mesh density, confirming convergence. The mesh distribution is illustrated in Figure S.1.

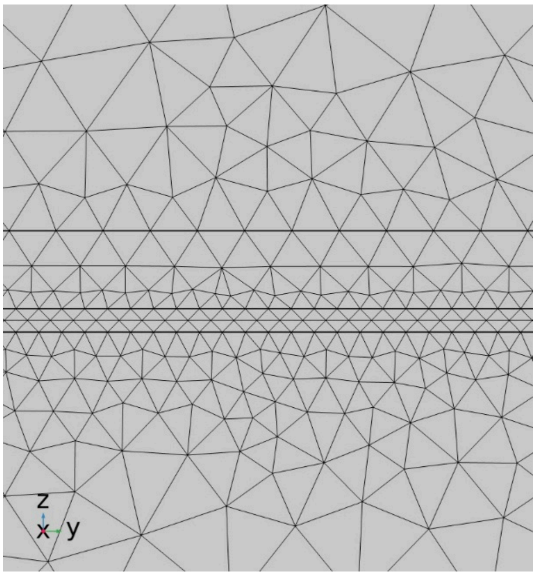

Figure S1. Non-uniform mesh distribution used in the simulations. A highly refined mesh is applied in regions with strong field variation, particularly near the graphene layer and the electric double layer (EDL), with gradually reduced density elsewhere. A convergence study confirms negligible variation beyond a certain mesh density.

Simulation Parameters:

A complete set of simulation parameters, including material properties, geometrical dimensions, and physical constants, has been added in Table 2. Additional details are provided in the Supplementary Information (Change 15).

Table S1. Complete set of simulation parameters, including material properties, geometrical dimensions, and physical constants.

Parameter	Value	Description
T	300[K]	Temperature
ε	78.5	relative permittivity of the electrolyte
ε_r	11.5	relative permittivity in stern layer
PH	7	Electrolyte PH
n_0	1.75e11[$\frac{1}{cm^2}$]	Intrinsic charge carrier density of graphene
V_f	1e6 [m/s]	Fermi velocity
D	0.0135[Å ^{2}/ps]	Protein self-diffusion
